# Invasion and diversity in *Pseudomonas aeruginosa* urinary tract infections

**DOI:** 10.1099/jmm.0.001458

**Published:** 2022-03-11

**Authors:** JN Newman, RV Floyd, JL Fothergill

**Affiliations:** ^1^​ Institute of Infection, Veterinary and Ecological Sciences, University of Liverpool, Liverpool, UK; ^2^​ School of Life Sciences, University of Liverpool, Liverpool, UK

**Keywords:** Urinary tract infection, antibiotic resistance, invasion, heterogeneity, intracellular

## Abstract

**Introduction.** *P. aeruginosa* is an opportunistic Gram-negative pathogen frequently isolated in urinary tract infections (UTI) affecting elderly and catheterized patients and associated with ineffective antibiotic treatment and poor clinical outcomes.

**Gap statement.** Invasion has been shown to play an important role in UTI caused by *E. coli* but has only recently been studied with *P. aeruginosa *. The ability of *P. aeruginosa* to adapt and evolve in chronic lung infections is associated with resistance to antibiotics but has rarely been studied in *P. aeruginosa* UTI populations.

**Aim. **We sought to determine whether phenotypic and genotypic heterogeneity exists in *P. aeruginosa* UTI isolates and whether, like urinary pathogenic *Escherichia coli *, these could invade human bladder epithelial cells – two factors that could complicate antibiotic treatment.

**Methodology.** *P. aeruginosa* UTI samples were obtained from five elderly patients at the Royal Liverpool University Hospital as part of routine diagnostics. Fourty isolates from each patient sample were screened for a range of phenotypes. The most phenotypically diverse isolates were genome sequenced. Gentamicin protection assays and confocal microscopy were used to determine capacity to invade bladder epithelial cells.

**Results.** Despite significant within-patient phenotypic differences, no UTI patient was colonized by distinct strains of *P. aeruginosa *. Limited genotypic differences were identified in the form of non-synonymous SNPs. Gentamicin protection assays and confocal microscopy provided evidence of *P. aeruginosa *’s ability to invade bladder epithelial cells.

**Conclusions.** Phenotypic variation and cell invasion could further complicate antibiotic treatment in some patients. More work is needed to better understand *P. aeruginosa* UTI pathogenesis and develop more effective treatment strategies.

## Background

Urinary tract infections (UTIs) are the most common healthcare acquired infections (HAIs) and cost an estimated $1.6 billion annually in the United States alone [1, 2]. *Pseudomonas aeruginosa* is an opportunistic Gram-negative bacteria frequently isolated as a cause of HAIs [1]. *P. aeruginosa* UTIs are associated with high levels of morbidity and mortality in elderly hospital patients [3, 4]. *P. aeruginosa* has been highlighted as an antibiotic resistant pathogen of the highest concern by the World Health Organization (WHO). These UTI isolates frequently display higher levels of antibiotic resistance than *Escherichia coli* – the most common UTI pathogen [5]. Uropathogenic *E. coli* (UPEC) research has found that UPEC can invade bladder epithelial cells (where it is protected from many antibiotics and the host immune system), form quiescent intracellular reservoirs (QIRs), and cause later episodes of recurrent UTI [6]. Elderly patients suffering from *P. aeruginosa* UTIs also experience recurrence after antibiotic treatment [4]. Existing literature has shown that *P. aeruginosa* can invade corneal cells, lung epithelial cells, and recently urinary epithelial cells [7–9].

*P. aeruginosa* is renowned for causing morbidity and mortality in lung infections of cystic fibrosis (CF) patients. Research has shown that *P. aeruginosa* communities evolve and diversify in the CF lung and this diversity makes them extremely difficult to eradicate [10, 11]. Current methods for assessing the antibiotic susceptibility of UTI pathogens utilize single colonies from urine samples. This may dramatically underestimate the diversity and therefore resistance of a particular pathogen causing a UTI and lead to inadequate treatment. Single nucelotide polymorphisms (SNPs) in resistance genes could confer antibiotic resistance to one isolate when it is lacking in another from the same sample. Furthermore, the existence of genetic diversity in a population can increase the likelihood that it can adapt and survive when challenged with antibiotics. Fully understanding the diversity of *P. aeruginosa* UTI communities and where they reside in the urinary tract will lead to more appropriate testing and treatment. Current evidence suggests that patients suffering from a *P. aeruginosa* UTI will be infected by a single clone type rather than multiple distinct strains [12]. We aim to identify whether phenotypic/genotypic heterogeneity exist in *P. aeruginosa* UTI and whether *P. aeruginosa* can invade human bladder epithelial cells.

## Methods

### Bacterial strains

*P. aeruginosa* strains PAO1 and PA14 were used as references [13]. *Escherichia coli* UTI89 was obtained from Rachel Floyd at the Liverpool Women’s Hospital [14]. Urine samples were collected and cultured for routine clinical diagnostics by the Royal Liverpool University Hospital from mid-stream urine samples plated on BD Chrom Orientation agar where *P. aeruginosa* was identified by MALDI-TOF as being the only pathogen present. Following growth of the bacteria from the BD chrom orientation agar plates, the bacteria were studied as an extension. No patient urine was collected. Multiple clinical isolates from a single UTI patient were streaked on Pseudomonas selective agar (Oxoid) and frozen stocks were created for 40 distinct colonies per UTI sample. Pooled urine from equal numbers of healthy male and female donors was filter sterilised and used as part of the project.

### Antibiotic susceptibility test

Colonies were picked from overnight growth on LB agar and adjusted to a 0.5 McFarland standard (OD600 of 0.08–0.12) in a 0.85% (w/v) sodium chloride solution. Adjusted cultures were spread on Mueller-Hinton agar (Sigma, 70192) plates and treated with antibiotic discs (Oxoid) containing gentamicin (10 µg), meropenem (10 µg), ciprofloxacin (5 µg) and piperacillin/tazobactam (30/6 µg) for 16 h at 37 °C. Zone diameters were measured and referenced to EUCAST breakpoints for sensitivity and resistance [15].

### Hypermutability assay

Hypermutability was tested by inoculating LB agar containing 300 μg ml^−1^ rifampicin with 10 µl of ~10^9^ c.f.u. ml^−1^ overnight cultures at 37 °C for 24 h. PAO1 and PAO1∆mutS (a known hypermutator) were used as negative and positive controls, respectively.

### Auxotrophy assay

Minimal growth assays were performed to check if urinary isolates exhibited auxotrophy. Isolates were streaked on M9 minimal media and grown for 24 to 48 h. Lack of growth at 48 h was taken as evidence of auxotrophy.

### Pyocyanin assay

Isolates were grown overnight in 5 ml LB broth shaking at 180 r.p.m. at 37 °C. Isolates were vortexed and aspirated to encourage oxygenation of pyocyanin before centrifugation at 14000 r.p.m. for 2 min. Then 200 µl of supernatant were transferred to 96-well plates and the absorbance was read at 695 nm with a BMG Labtech Fluostar Omega plate reader. Absorbance values were normalized by subtraction of LB A695 values. Isolates with normalized A695 values exceeding 0.1 were classified as pyocyanin overproducers [16].

### Statistical analyses

Statistical analyses were conducted in GraphPad Prism five software. Histograms were generated to check data for normal distributions. If data were normally distributed, parametric tests, including a One-way analysis of variance (ANOVA), and Tukey post-hoc tests were used to identify pairwise significant differences.

### Random Amplification of Polymorphic DNA (RAPD) PCR

Isolates were boiled at 100 °C for 5 min in 100 µl nuclease free water (Sigma, H20MB) to extract genomic DNA. Random amplification of polymorphic DNA (RAPD) PCR was carried out using primer 272 (Sigma, AGCGGGCCAA) and genomic DNA. Then 20 µl of RAPD products was mixed with 4 µl 6× purple loading dye (New England Biolabs) were separated by gel electrophoresis in 1.5% agarose gels with 0.5× TBE running buffer for 3 h at 70V.

### DNA extraction and sequencing of ten clinical isolates

The ten clinical isolates from five patients that were selected for further study were grown over night shaking at 180 r.p.m. and 37 °C. DNA was extracted with the Promega Wizard Genomic DNA Purification Kit and protocol. DNA was rehydrated in DNase-free RNase-free sterile water and stored at 4 °C until sequencing. DNA was Illumina sequenced by the Centre for Genomic Research (CGR) at the University of Liverpool using paired end reads (ENA primary accession number PRJEB43148). The raw Fastq files were trimmed for the presence of Illumina adapter sequences using Cutadapt version 1.2.1. The option -O three was used, so the 3' end of any reads which match the adapter sequence for 3 bp or more are trimmed. The reads were further trimmed using Sickle version 1.200 with a minimum window quality score of 20. Reads shorter than 20 bp after trimming were removed.

### Pairwise SNP variant analysis using BactSNP

SNP variant analysis was conducted using the BactSNP programme https://github.com/IEkAdN/BactSNP [17]. BactSNP was run on Linux Ubuntu for Windows version 18.04 LTS. Pairs of isolates from each patient were assembled against either PAO1 (GCF_000006765.1) or PA14 (GCF_000014625.1) depending on which reference genome they were most similar to. BactSNP was run with default arguments where SNP variants are only called if the coverage at a base is greater than ten and more than five bases away from a suspected INDEL.

### Culture methods of cell lines

Cryopreserved 5637 (ATCC, HTB-9) cells were thawed rapidly in a 37 °C water bath and seeded in 7 ml of prewarmed complete medium in a T25 cell culture flask. Complete medium consisted of 10% feotal bovine serum (FBS) (Life Technologies, 10270106), 5 ml of 100× penicillin-streptomycin (Sigma, P0781) dissolved in 500 ml Rosa Parks Memorial Institute (RPMI) medium with l-glutamine and sodium bicarbonate (Sigma, R8758). Media was replaced the following day. Cells were detached and re-seeded in T75 flasks at ~90% confluency. The 5637 cells were used for a maximum of ten passages from ATCC stock vials between passage 41 and 51. Flasks were split upon reaching 80–90% confluency. Prior to splitting, media was removed from cells and cells were washed with 10 ml of Dulbecco’s PBS (DPBS) (Life Technologies, 14200083) +0.1% EDTA per T75. Cells were incubated with 2 ml of trypsin-EDTA (Sigma, T3924; 0.5 g porcine trypsin and 0.2 g EDTA per litre Hank’s Balanced Salt Solution) per T75 for 10 min. Flasks were gently horizontally tapped against bench to detach cells. Then 10 ml of RPMI +10% FBS was added to each T75 to inactivate the trypsin. From this, 2 ml of detached cells (~2×10^6^) were seeded into fresh T75 flasks with 14 ml of RPMI +10% FBS.

### Gentamicin protection assays

#### Invasion in 12-well plates with clinical isolates

Gentamicin protection assays were performed to quantify the ability of bacteria to adhere to, invade and persist within bladder epithelial cells. We utilised 12-well cell culture plates (Starlab, CC7682) which were seeded with 2×10^5^ bladder urothelial cells (5637 ATCC) in complete medium and grown for 48 h to form a confluent monolayer. Cell supernatant was removed at 24 h to remove FBS and penicillin-streptomycin and replaced with 1000 µl of RPMI 1640. Bacteria were grown overnight in LB broth, pelleted at 5000 ***g*** for 5 min and resuspended in RPMI. Bacteria were adjusted to an OD600 of 0.5 before dilution in RPMI to create an evenly distributed bacteria:media mastermix for infection of cell monolayers at a multiplicity of infection (MOI) of ~25. Cell monolayers were washed with DPBS with calcium and magnesium (DPBS++) (Life Technologies, 14040174) and infected with 990 µl of the appropriate bacteria:RPMI mastermix before incubation at 37 °C for 2 h. At 2 h post-infection (hpi) 10 µl of 10% v/v Triton X-100 was added to one set of triplicate wells while another set of triplicate wells had the supernatant removed, was washed 3× with PBS++ and cells were lysed with 1000 µl of 0.1% Triton X-100. Plates were left on ice for 5–10 min, scraped, serially diluted and then plated on to LB agar for colony forming units per ml (c.f.u. ml^-1^) determination. These two conditions are termed ‘Total’ and ‘Bound’, respectively. The ‘Total’ condition represents bacterial numbers found in the extracellular supernatant, bound to the cells and intracellularly. The ‘Bound’ condition represents bacterial numbers found bound to cells and intracellularly. At 2 hpi supernatant was removed from remaining wells, wells were washed twice with DPBS ++ and 1000 µl of 200 µg ml^−1^ gentamicin in RPMI was added to wells. Gentamicin is a membrane impermeable antibiotic and therefore kills all extracellular bacteria while preserving intracellular bacteria. Intracellular bacteria counts were obtained at 4 hpi by removing gentamicin from wells, washing twice with PBS++, lysing with 1000 µl of 0.1% Triton X-100 and plating serial dilutions on LB agar.

Prior to infection of 12-well cell culture plates seeded with human bladder cells, wells were seeded in triplicate with experimental quantities of the *P. aeruginosa* strains to be tested in cell culture media. Strains were incubated for 2 h at 37 °C, washed with PBS, and treated with 0, 100, and 200 µg ml^−1^ gentamicin in RPMI for 2 h to prior to colony forming unit enumeration to ensure that all strains were susceptible. No strains showed evidence of survival at 100 or 200 µg ml^−1^ gentamicin in RPMI, but 200 µg ml^−1^ gentamicin in RPMI was selected for extra precaution.

### Invasion in cell culture inserts for fluorescent imaging

Invasion assays were conducted in cell culture inserts (Sigma, PIHP01250) with PAO1 in 24-well plates. Cell culture inserts seeded with 2×10^5^ 5637 cells were grown for 24 h to reach confluency. PAO1 was grown overnight in LB broth at 37 °C in a shaking incubator. PAO1 was pelleted at 5000 ***g*** for 5 min. LB supernatant was poured off and replaced with RPMI (without antibiotics) for infection of 5637 cells. Bacteria culture was adjusted to an OD600 of 0.5 and 200 µl of adjusted culture was added to 19.8 ml of RPMI. Cell culture inserts were washed once with PBS++ then 400 µl of diluted PAO1 culture was added to each cell culture insert (MOI ~25). Plates were incubated for 3 h at 37 °C to allow bacteria to adhere to and invade epithelial cells. Media was removed from inside and outside the cell culture inserts and replaced with RPMI supplemented with 200 µg ml^−1^ gentamicin. Plates were then incubated for an additional hour while the gentamicin killed off all extracellular bacteria. The cell culture inserts were washed two times with PBS++ before addition of 400 µl of 4% methanol free formaldehyde (Fisher Scientific, R37814) to fix the cells and bacteria prior to staining. Cells were fixed for 10 min at room temperature before proceeding to the fluorescent staining protocol.

### Fluorescent staining protocol

Formaldehyde was removed from fixed cells in cell culture inserts and discarded in hazardous waste. Cell culture inserts were washed twice with PBS. Cell culture inserts were quenched in cold 150 mM glycine in PBS for 15 min to quench background fluorescence and then washed with PBS three times. Cell culture inserts were blocked in blocking solution (5% FBS, 2.5% cold fish skin gelatin, 0.1% v/v Triton X-100, 0.05% v/v Tween-20) for 1 h at room temperature. A 1 : 500 dilution of primary antibodies (Abcam, Ab74980) in antibody solution (2.5% FBS, 1.25% cold fish skin gelatin, 0.1% v/v Triton X-100, 0.05% v/v Tween-20) was added to cell culture inserts before overnight incubation at 4 °C. Antibodies were removed and washed four times in PBS. Secondary antibody (goat anti-chicken conjugated to AlexaFluor-488) diluted 1:1000 in antibody solution was added for 1 h at room temperature protected from light. Cell culture inserts were washed once with PBS before addition of a dual-labelling solution of DAPI (1 ug ml^−1^) and AlexaFluor-633-conjugated phalloidin (1.65 uM) for 1 h at room temperature protected from light. Finally, cell culture inserts were washed twice with PBS and then cut out with a scalpel, mounted on a glass microscopy slide with Prolong Gold Antifade Reagent (Life Technologies, P36930) and a glass coverslip. Slides were imaged with LSM710 confocal microscope and images were taken by the Centre for Confocal Microscopy (CCI) at the University of Liverpool.

## Results

### Clinical *P. aeruginosa* UTI isolates demonstrate within-patient phenotypic diversity

We characterized the phenotypes of 200 *P. aeruginosa* isolates from five UTI patients with antimicrobial susceptibility tests (ASTs) (Figs S1–S4, available in the online version of this article), pyocyanin assays (Fig. S5), hypermutability assays, and auxotrophy assays (Table 1). No evidence of hypermutability or auxotrophy was found but significant phenotypic heterogeneity was identified in ASTs and pyocyanin assays (Figs S1–S5). We selected 2/40 of the most phenotypically different isolates from each of the five patient samples for further genotypic comparison. We found significant differences (Tukey post-hoc test, *P*<0.01) between isolates from a single patient in the cases of patient samples A, G and J, but not from patient samples B and D (Table 1).

**Table 1.** Phenotypic traits of 10 clinical isolates selected for further study. Two of the most phenotypically variable isolates were selected from each of the five patients. Originally, 40 isolates were selected from each patient’s urine culture (isolate A39 is the 39th isolate from patient A)

Isolate	Ciprofloxacin AST	Gentamicin AST	Meropenem AST	Piperacillin/Tazobactam AST	Pyocyanin assay	RAPD PCR typing	ExoU/S
A1	S	S	S*	S*	overproducer	A type	ExoU
A39	S	S	S*	S*	underproducer	A type	ExoU
B12	S	S	R	S	overproducer	B type	ExoU
B13	R	S	R	S	underproducer	B type	ExoU
D1	S	S	S	S	overproducer	D type	ExoS
D35	S	S	S	S	overproducer	D type	ExoS
G7	R*	S	R	S*	underproducer*	G type	ExoS
G30	S*	S	R	S*	overproducer*	G type	ExoS
J7	S*	S*	S	S	underproducer	J type	ExoS
J28	S*	S*	S	S	underproducer	J type	ExoS

*Indicates significant difference between two isolates from the same patient (One-way ANOVA Tukey post-hoc, *P*<0.01).

### Degree of within patient phenotypic diversity correlates with number of non-synonymous SNPs

SNP variant calling was performed between pairs of isolates from single patients that appeared to be the most phenotypically diverse (Table 2) with BactSNP [17]. Isolates A1 and A39 had two SNP variants between them (Table 2). A39 had a non-synonymous SNP that varied from A1 and the PA14 reference in the *mexR* efflux pump repressor gene. A1 had a non-synonymous SNP in the *mexS* efflux repressor gene that differed from A39 and PA14. No SNP variants were identified between the pairs of isolates from patients B and D and these isolates also had no statistically different phenotypic differences (Table 1). Isolates G7 and G30 had five SNP variants between them. G7 had non-synonymous SNPs that differed from G30 and PAO1 in *lasR*, *parS* and *nalD*. G30 had a stop codon truncation SNP in the *pvdQ* gene and a SNP in the *clpB* gene. J7 and J28 had two non-synonymous SNPs that differed between them. J7 had a SNP in *metH*. J28 had an SNP in *rmlB*.

**Table 2.** Single nucleotide polymorphisms (SNPs) identified between pairs of isolates from patients A, G and J using BactSNP analysis. Isolates from patients B and D had no SNP variants. PAO1 or PA14 were used as reference genomes for *ExoS*-type and *ExoU*-type isolates, respectively.

SNPs in A1 vs A39							
Location	Gene affected	PA14	A1	A39	Amino acid substitution	Type	Gene function
486253	*mexR*	C	C	T	Arg>His	missense	efflux pump repressor
2820706	*mexS*	T	C	T	Cys>Arg	missense	efflux pump repressor
**SNPs in G7 vs G30**							
**Location**	**Gene affected**	**PAO1**	**G7**	**G30**	**Amino acid substitution**	**Type**	**Gene function**
1558831	*lasR*	G	A	G	Val>Met	missense	quorum sensing
1951314	*parS*	C	T	C	Ala>Thr	missense	two-component system
2638491	*pvdQ*	C	C	T	Trp*	stop gained	quorum quenching and iron response regulator
4006879	*nalD*	G	T	G	Glu*	stop gained	Efflux pump repressor
5093201	*clpB*	G	G	A	Upstream variant	N/A	Chaperone protein linked to stress tolerance
**SNPs in J7 vs J28**							
**Location**	**Gene affected**	**PAO1**	**J7**	**J28**	**Amino acid substitution**	**Type**	**Gene function**
2003070	*metH*	C	G	C	Arg>Pro	missense	methionine synthesis and swarming regulation
5810918	*rmlB*	T	G	T	Leu>Arg	missense	dTDP-rhamnose biosynthesis

### *P. aeruginosa* is capable of invading human bladder epithelial cells *in vitro*

**Fig. 1.**
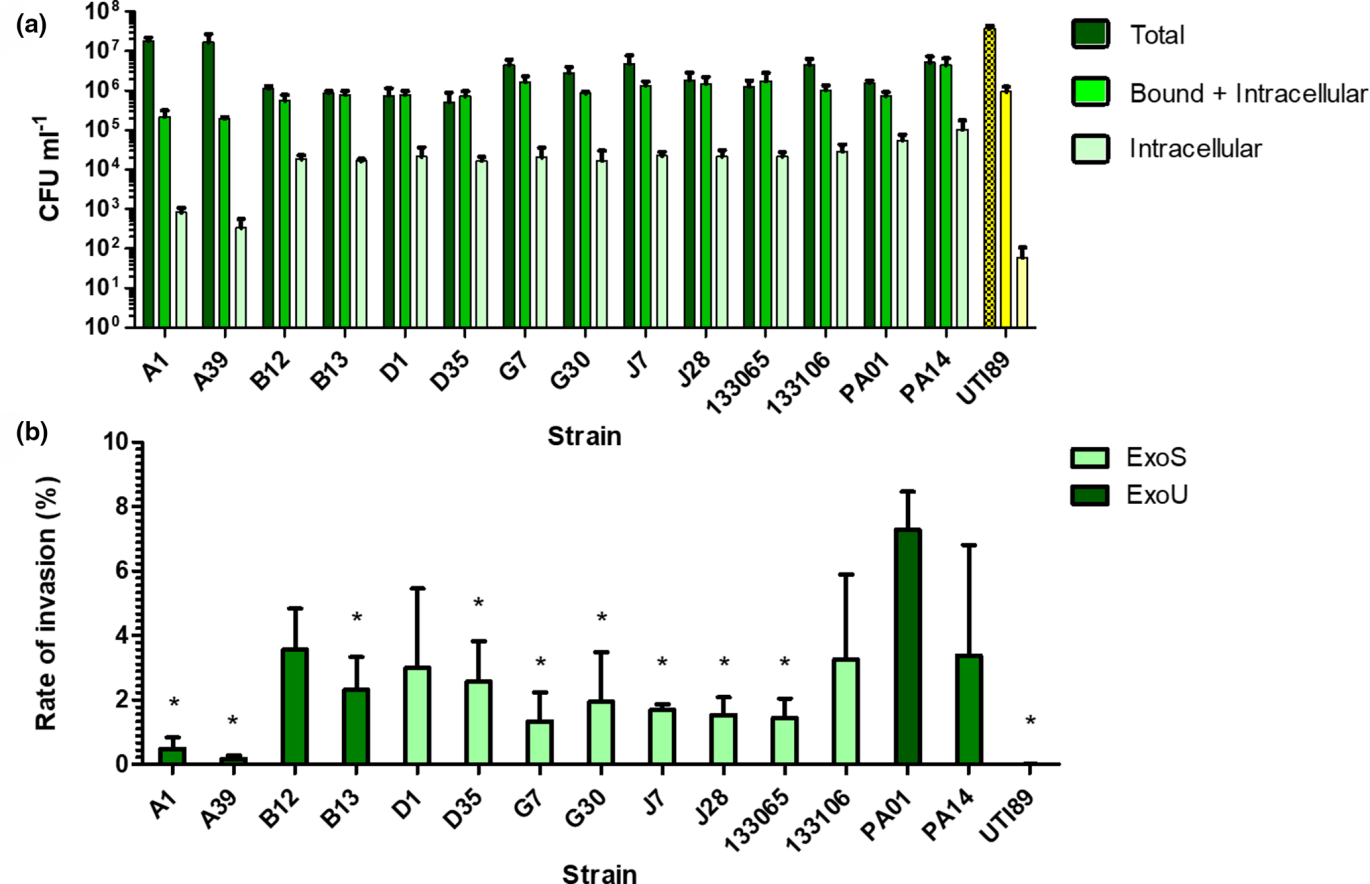
(**a**) Quantification of bacterial strains at different time points of an *in vitro* invasion assay in 5637 human bladder epithelial cells. ‘Total’ values represent intracellular bacteria, adherent bacteria and non-adherent bacteria in the cell supernatant isolated at 2 h post-infection (hpi). ‘Bound+Intracellular’ values represent adherent and intracellular bacteria isolated at 2 hpi. ‘Intracellular’ values represent intracellular bacteria isolated at 4 hpi following treatment with 200 ug ml^−1^ gentamicin to eliminate extracellular bacteria. PAO1 and PA14 are lab strains of *P. aeruginosa *. UTI89 is a commonly studied strain of *E. coli* isolated from a urinary tract infection shown in yellow. All other strains are clinical UTI isolates of *P. aeruginosa* (*n*=3 for all strains). (**b**) Invasion rates of bacterial strains at different time points of an *in vitro* invasion assay in 5637 human bladder epithelial cells. Invasion rate is calculated by dividing the number of intracellular bacteria isolated at 4 h post-infection by the number of adherent and intracellular bacteria isolated at 2 h post-infection. Light green bar indicates strain contains *exoS* gene, Dark green bar indicates strain contains *exoU* gene. * Indicates an isolate’s mean rate of invasion is significantly different from PAO1’s (Tukey post-hoc test *P*<0.05). (*n*=3 for all strains).

We performed gentamicin protection assays to quantify the ability of bacteria to adhere to and invade bladder epithelial cells. The highest quantity of ‘Bound +Intracellular’ bacteria were recovered from cells infected with PA14 (4.43×10^6^ c.f.u. ml^−1^) (Fig. 1a). The lowest quantities of *P. aeruginosa* ‘Bound +Intracellular’ bacteria were recovered from clinical isolates A1 (2.18×10^5^ c.f.u. ml^−1^) and A39 (1.97×10^5^ c.f.u. ml^−1^). PA14 had significantly higher levels of ‘Bound +Intracellular’ bacteria than every other strain (Tukey post-hoc test *P*<0.05). No other strains differed significantly from each other.

The highest quantity of ‘Intracellular’ bacteria was also recovered from cells infected with PA14 (1.04×10^5^ c.f.u. ml^−1^) (Fig. 1a). The lowest quantity of *P. aeruginosa* ‘Intracellular’ bacteria was recovered from cells infected with A1 (8.2×10^2^ c.f.u. ml^−1^) and A39 (3.36×10^2^ c.f.u. ml^−1^). Among *E. coli* strains, UTI89 (6.00×10^1^ c.f.u. ml^−1^) had the lowest quantity of ‘Intracellular’ bacteria recovered. PA14 infected cells had the highest levels of ‘Intracellular’ bacteria.

**Fig. 2.**
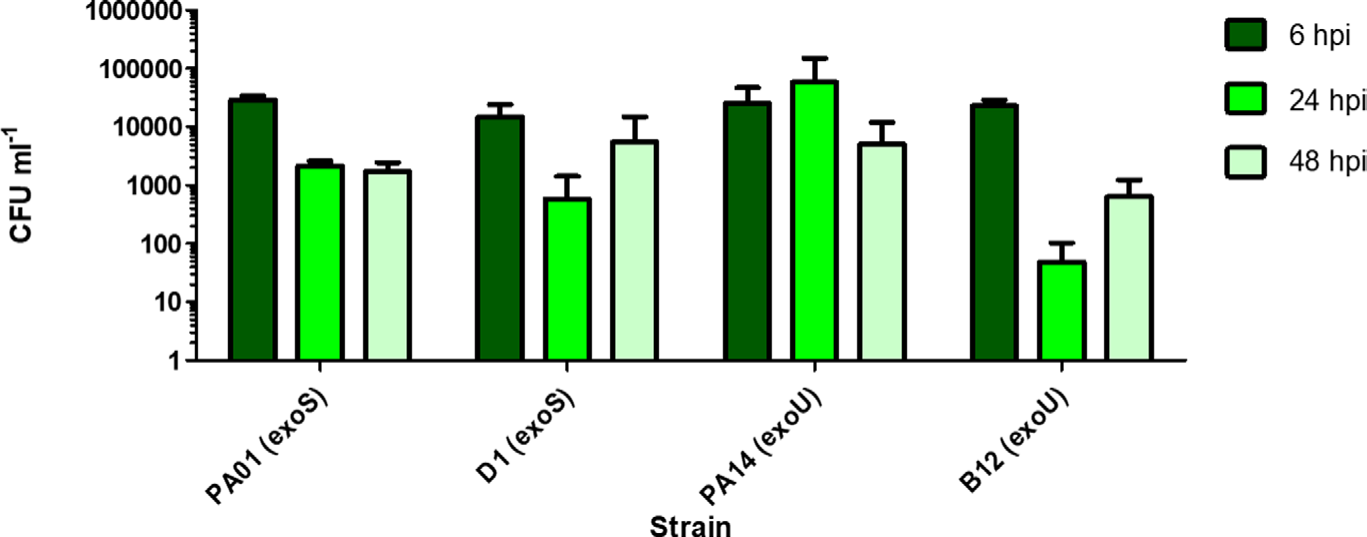
Quantification of intracellular bacteria recovered at 6, 24 and 48 h post-infection (hpi) after treatment with 200 µg ml^−1^ gentamicin. PAO1 and D1 are ExoS-type strains while PA14 and B12 are ExoU-type strains. There were no significant differences in quantities of intracellular bacteria recovered for different strains at the same time point (*n*=3 for each strain, error bars represent 1 SD of the mean).

Invasion rates were calculated for every strain by dividing the number of intracellular bacteria at 4 hpi by the number of ‘Bound +Intracellular’ bacteria at 2 hpi and multiplying by 100 to express as a percentage (Fig. 1b). PAO1 had the highest mean invasion rate, with 7.28% of ‘Bound’ bacteria being recovered at the ‘Intracellular’ time point. A Tukey post-hoc test revealed that PAO1 (7.28%) had a significantly higher (*P*<0.05) invasion rate than A1 (0.48%), A39 (0.17%), B13 (2.31%), D35 (2.56%), G7 (1.33%), J7 (1.67%), J28 (1.53%), 133 065 (1.44%), and UTI89 (0.01%). PAO1 did not invade at a significantly higher rate than PA14 (3.37%), B12 (3.57%), D1 (2.99%), G30 (1.95%) or 133 106 (3.26 %). At 48 h invasion assays of ExoU-producing isolates (PA14 and B12) vs ExoS-producing isolates (PAO1 and D1) revealed no significant differences in their abilities to persist intracellularly over time (Fig. 2).

We also managed to significantly reduce the number of intracellular bacteria recovered by performing gentamicin protection assays with cell-permeable ofloxacin (Fig. S8).

### Bladder epithelial cells infected by ExoU-producing *P. aeruginosa* strains produce significantly higher levels of IL-1β than cells infected by ExoS-producing strains at 4 hours post-infection

**Fig. 3.**
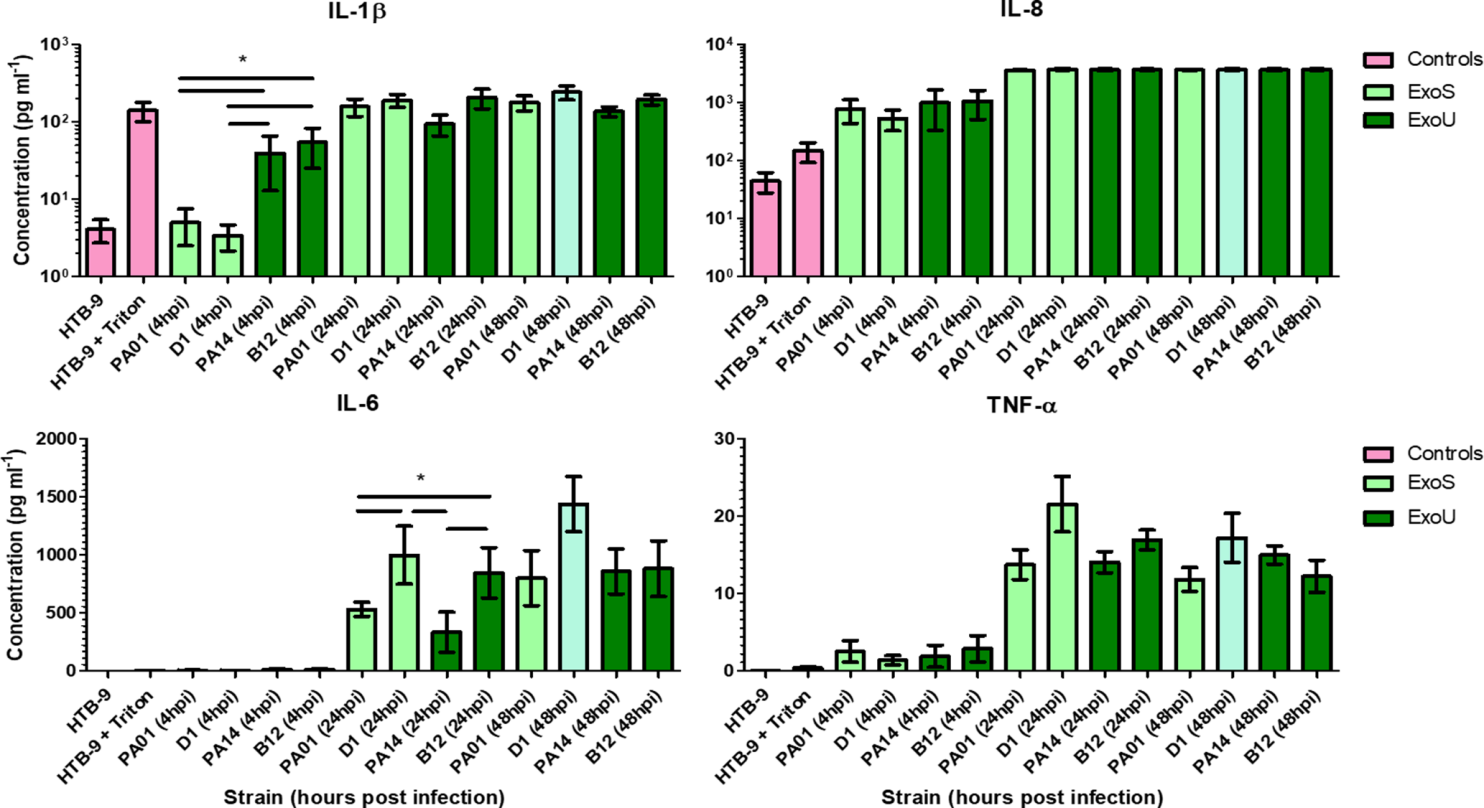
Levels of cytokines and pro-inflammatory molecules detected in cell supernatants using the MSD V-plex assay. The 5637 (HTB-9) cells were infected with ExoS-type (PAO1, **D1**) and ExoU-type (PA14, **B12**) strains for 4, 24 and 48 h.

Supernatants were collected from 5637 cells infected by ExoS (PAO1 and D1) and ExoU (PA14 and B12) producing strains of *P. aeruginosa* and assayed for an array of eight cytokines and pro-inflammatory molecules to see if bladder epithelial cells would respond differently to different strains. Significantly higher levels of IL-6 were seen in cells infected by clinical isolates B12 (846 pg ul^−1^) and D1 (998 pg ul^−1^) when compared to the cells infected by PAO1 (530 pg ul^−1^) and PA14 lab strains (334 pg ul^−1^) at 24 h post-infection (One-way ANOVA, Tukey post-hoc, *P*<0.05) (Fig. 3). At 4 h post-infection, PA14 and B12 infected cells produced significantly higher levels (39 and 54 pg ul^−1^, respectively) of IL-1β than PAO1 and D1 infected cells (5 and 3 pg ul^−1^, respectively) (One-way ANOVA, Tukey post-hoc, *P*<0.05) (Fig. 3). No statistically significant differences were seen in production of GM-CSF, IL-10, IFN-γ, IL-17A, IL-8 and TNF-α (Figs 3 and S7).

### Confocal imaging confirms presence of intracellular *P. aeruginosa* in bladder epithelial cells

**Fig. 4.**
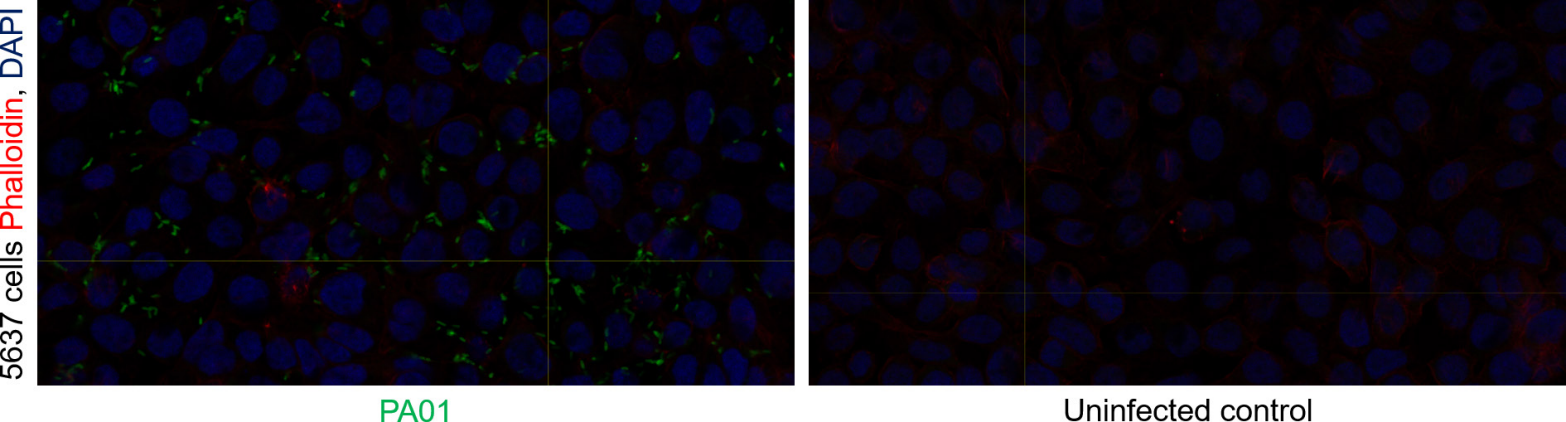
Confocal image of 5637 cells infected by PAO1 (**a**) and uninfected 5637 cells (**b**) showing a single Z-slice. The 5637 cells stained with DAPI (blue) and Phalloidin conjugated to AlexaFluor-647 (red). Also stained with anti-Pseudomonas Ab74980 primary antibodies and goat anti-chicken conjugated to AlexaFluor-488 secondary antibodies (green).

**Fig. 5.**
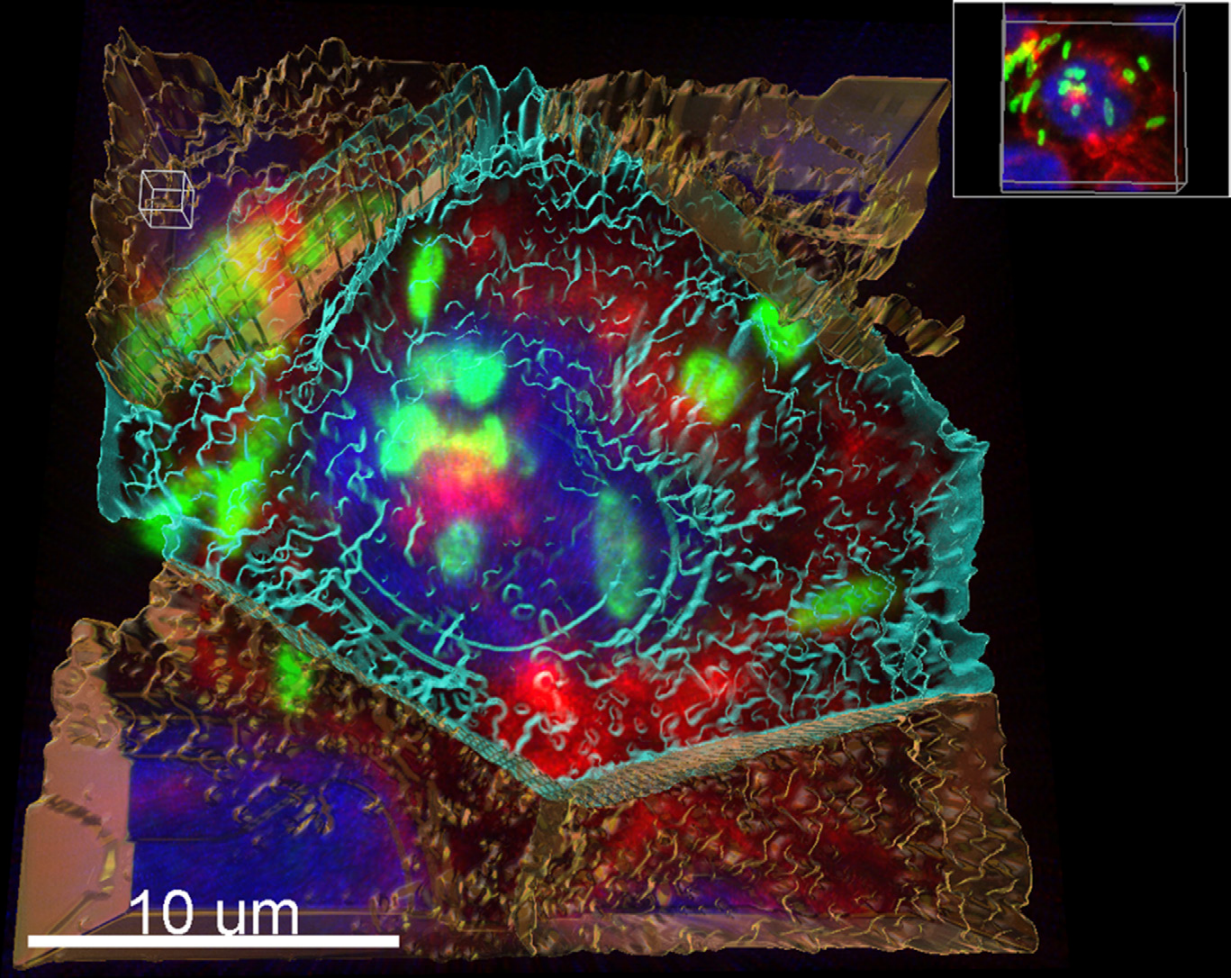
Confocal reconstruction of PAO1 (green) inside 5637 cells (red membranes with blue nuclei) 5637 cells stained with DAPI and Phalloidin conjugated to AlexaFluor-647. PAO1 stained with anti-Pseudomonas Ab74980 primary antibodies and goat anti-chicken conjugated to AlexaFluor-488 secondary antibodies.

Invasion assays were conducted with PAO1 infecting 5637 cells grown on cell culture inserts. Cells and bacteria were fixed with 4% formaldehyde before staining with anti-PAO1 antibodies to highlight intracellular bacteria and phalloidin/DAPI to show the human bladder cells. Stained cell culture insert filters were cut out and mounted on glass slides for confocal imaging. Images show evidence of intracellular bacteria in infected 5637 monolayers treated with gentamicin (Figs 4 and 5).

## Discussion

We sampled 40 *P *. *aeruginosa* isolates per patient from five elderly UTI patients and found significant phenotypic heterogeneity in 3/5 patient sample communities. Genotypic differences were found between pairs of isolates from the three patients where significant phenotypic heterogeneity was found. Isolate pairs from patients B and D had no significant differences in their phenotypes (Table 1) and had no pairwise SNPs (Table 2). Furthermore, paired isolates from patient G had the most phenotypic significant differences (significantly different ciprofloxacin resistance, piperacillin/tazobactam resistance and pyocyanin production) while also having the most pairwise SNPs (five, as seen in Table 2). This suggests that some of the significant phenotypic differences observed between pairs of isolates from patients A, G and J may have been the result of variations identified by SNP analysis. SNPs were identified in genes associated with quorum sensing, motility, biofilm formation and antibiotic resistance. The confirmation of within-patient genotypic/phenotypic diversity could have important implications for how we diagnose and treat *P. aeruginosa* UTIs. Existing genotypic/phenotypic diversity may drive evolutionary changes in the dynamic environment of the urinary tract. The presence of certain SNPs in a *P. aeruginosa* UTI population may predict the course of infection or determine the optimal treatment strategy.

We found no evidence of a patient being infected by multiple distinct strains of *P. aeruginosa *. Conversely, isolates descended from distinct lineages were found in the lungs of 7/24 bronchiectasis patients even though there is typically greater genotypic diversity of *P. aeruginosa* between patients than there is within a single patient [18]. This could signal that the lungs offer a greater variety of niches for diversification than the urinary tract. Studies of uropathogenic *E. coli* (UPEC) suggest that multiple lineages are rarely maintained in the urinary tract [19, 20]. Instead, a strain with higher fitness will displace other strains competing for the urinary tract niche [19]. Alternatively, this could highlight the inadequacy of urine samples for the identification of all possible strains inhabiting the urinary tracts of sampled patients. The samples in this study are representative of a portion of a UTI population at a single moment in time and tell us nothing about the stability of those population dynamics. Cottalorda *et al*. have started to shed light on how *P. aeruginosa* UTI populations may change over time [12]. As in this study, UTI patients were only ever inhabited by a single clonal type of *P. aeruginosa* but limited genotypic differences were found in the form of SNPs. Isolates from two separate patients developed convergent adaptations in genes encoding transcriptional regulators, two-component systems, and carbon compound catabolism [12]. More work is needed to see if these kinds of adaptations are typical of *P. aeruginosa* UTI populations. Also, further work is needed to understand spatiotemporal changes in *P. aeruginosa* heterogeneity where samples can be taken from different niches in the urinary tract similar to the work performed with *E. coli* in mouse models of UTI [21].

We have shown that *P. aeruginosa* can invade bladder epithelial cells in an *in vitro* model. The results of our gentamicin protection assays suggest that *P. aeruginosa* strains possess a widely conserved ability to invade 5637 cells and confirm the recent findings of Penaranda *et al*. [9]. Based on similar research in UPEC, it is likely that *P. aeruginosa* can exist as an intracellular pathogen during UTI in humans. There is extensive evidence that *P. aeruginosa* preferentially binds to, invades and injures wounded epithelium [22–26]. The 5637 cells are immortalized human bladder epithelial cells and form monolayers instead of the stratified layers of the bladder epithelium. After toxin-mediated killing of some cells, 5637 monolayers could easily become exposed to attack from vulnerable basolateral surfaces. Previous research has classified *P. aeruginosa* strains as either cytotoxic (ExoU-producing) or invasive (ExoS-producing) [7, 8]. However, we found no significant differences in levels of intracellular ExoS and ExoU strains over 48 h (Fig. 2). We did observe that ExoU-producing strains provoked a significantly stronger IL-1β response from 5637 cells at 4 hpi which could relate to the greater cytotoxicity of ExoU compared to ExoS.

The idea that *P. aeruginosa* has a widely conserved ability to invade vulnerable epithelial cells correlates well with the high prevalence of *P. aeruginosa* UTI in hospitalized elderly populations. Future treatment strategies for nosocomial UTI may rely less on eradicating pathogens like *P. aeruginosa* and more on maintaining barrier integrity of the human urinary tract.

## Supplementary Data

Supplementary material 1
