# Supplementary material for: Invasion and diversity in Pseudomonas aeruginosa urinary tract infections
Source: J Med Microbiol. 2022 Mar 11;71(3):001458. doi: 10.1099/jmm.0.001458 (PMC9176269; doi:10.1099/jmm.0.001458)
Supplement: Supplementary material 1 [file jmm-71-1458-s001.pdf]

Supplementary data

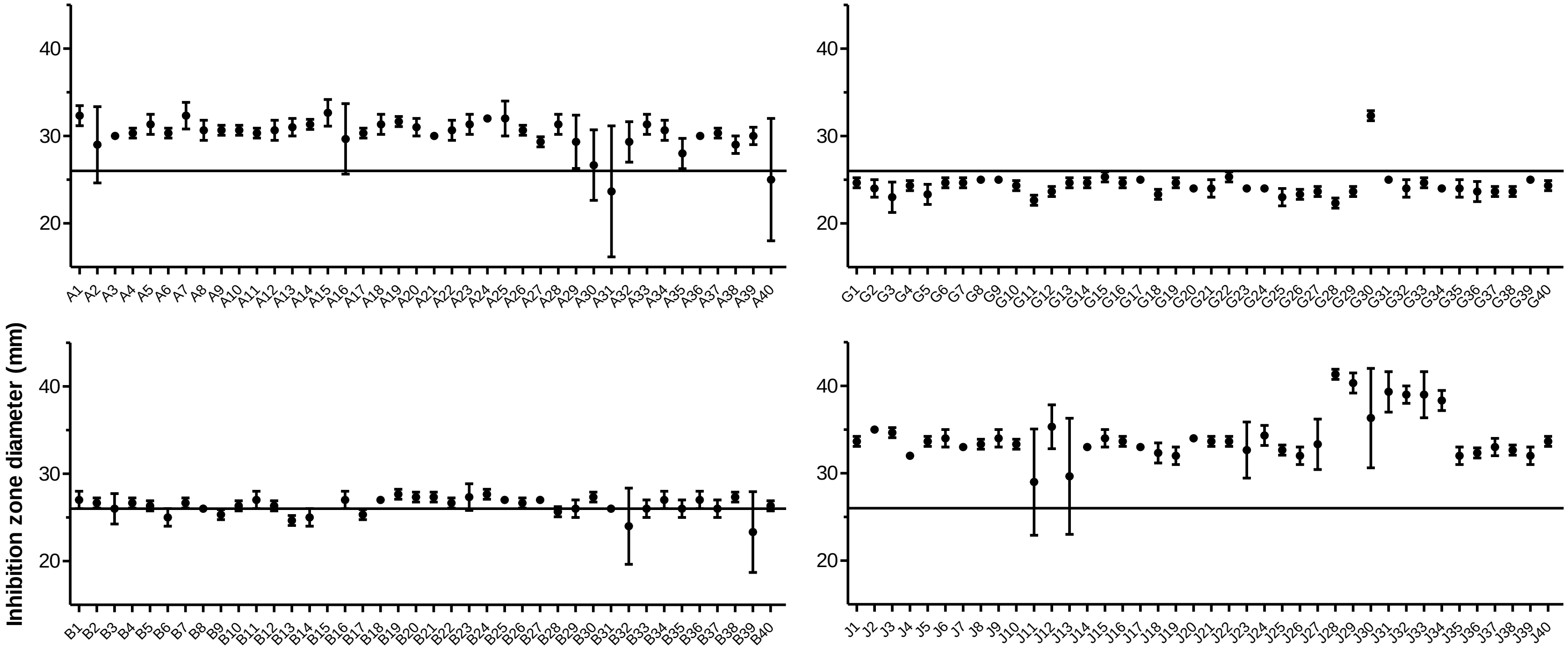

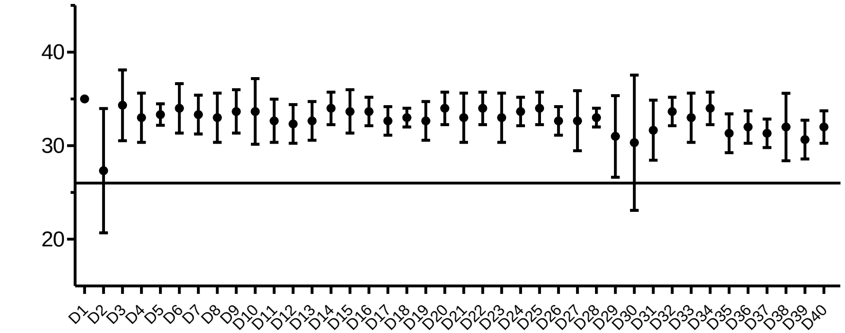

Figure S1. Ciprofloxacin antimicrobial susceptibility tests conducted with 200 clinical Pseudomonas aeruginosa UTI isolates from 5 patients (A, B, D, G and J). Test conducted according to EUCAST disk diffusion protocol with EUCAST resistance breakpoint indicated by solid black line. Error bars represent 1 SD of the mean, each isolate was tested 3 times.

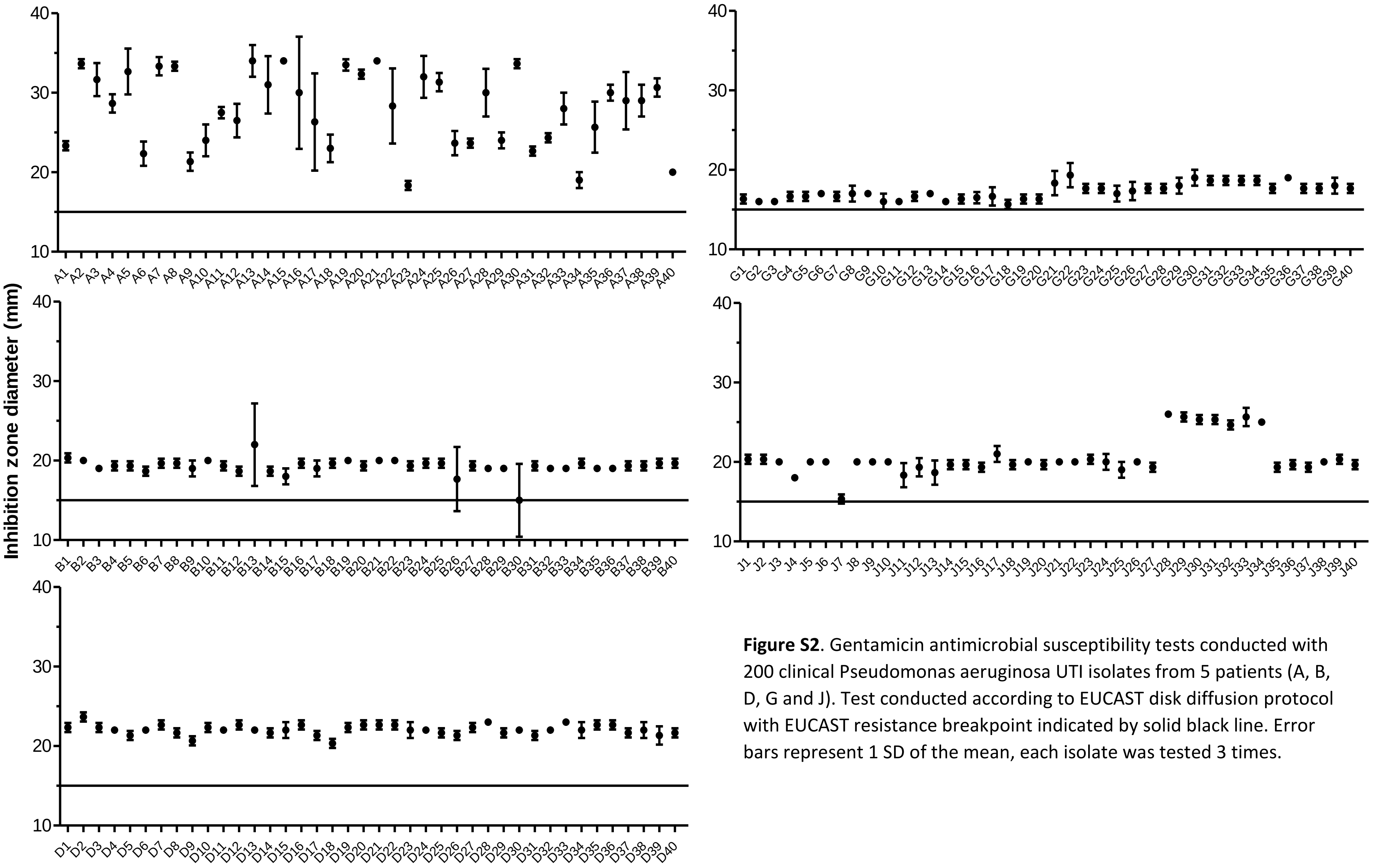

Figure S2. Gentamicin antimicrobial susceptibility tests conducted with 200 clinical Pseudomonas aeruginosa UTI isolates from 5 patients (A, B, D, G and J). Test conducted according to EUCAST disk diffusion protocol with EUCAST resistance breakpoint indicated by solid black line. Error bars represent 1 SD of the mean, each isolate was tested 3 times.

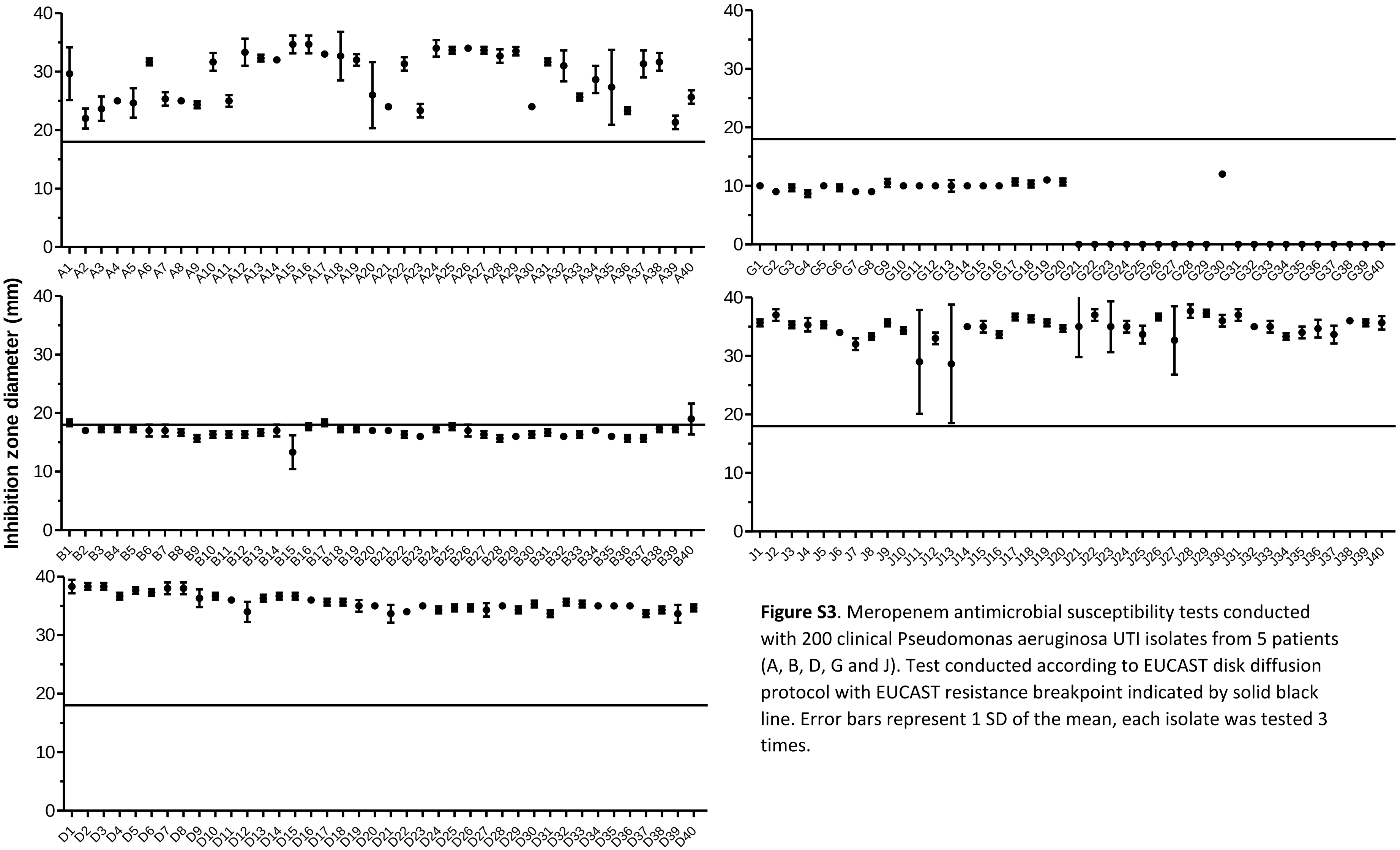

Figure S3. Meropenem antimicrobial susceptibility tests conducted with 200 clinical Pseudomonas aeruginosa UTI isolates from 5 patients (A, B, D, G and J). Test conducted according to EUCAST disk diffusion protocol with EUCAST resistance breakpoint indicated by solid black line. Error bars represent 1 SD of the mean, each isolate was tested 3 times.

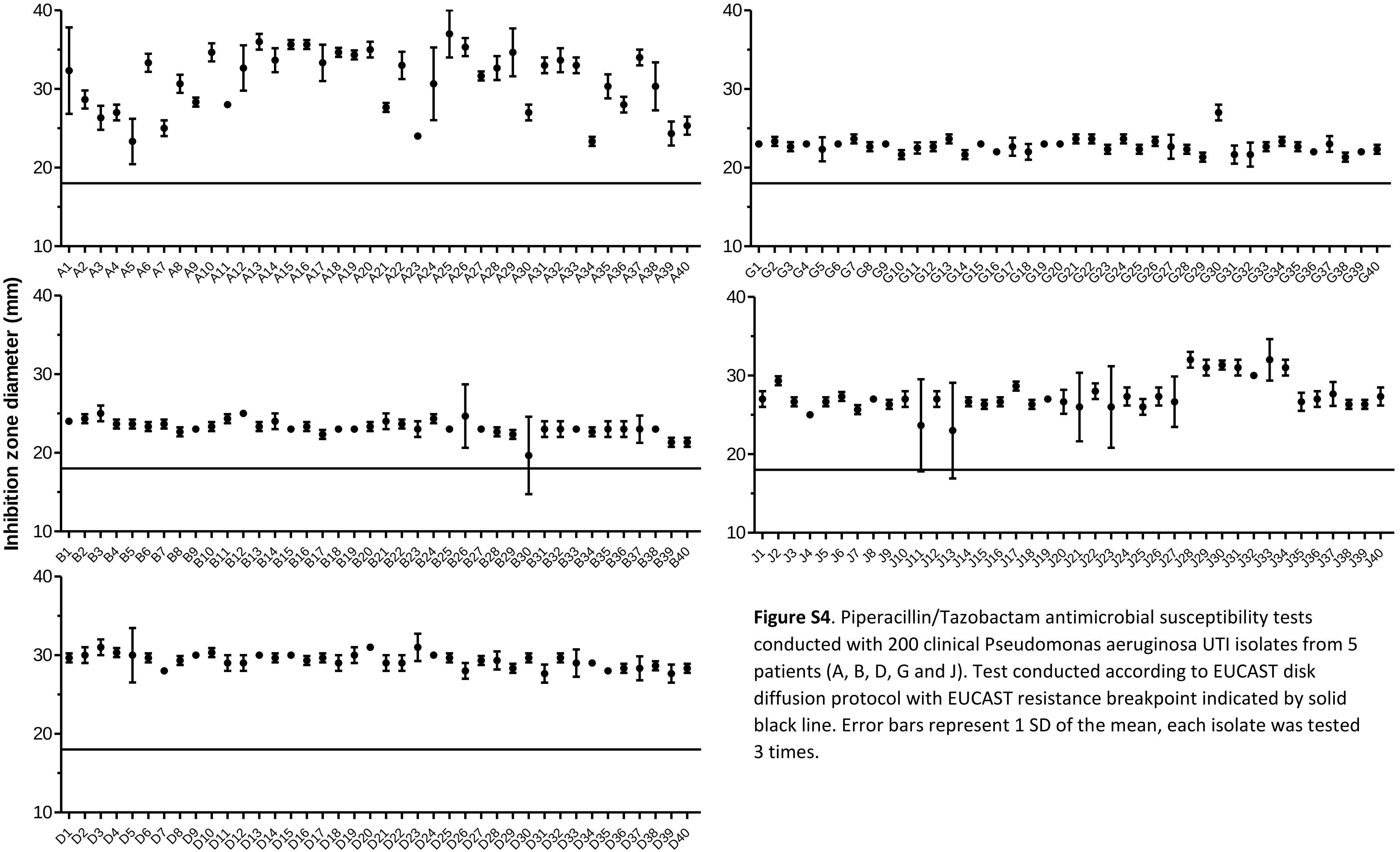

Figure S4. Piperacillin/Tazobactam antimicrobial susceptibility tests conducted with 200 clinical Pseudomonas aeruginosa UTI isolates from 5 patients (A, B, D, G and J). Test conducted according to EUCAST disk diffusion protocol with EUCAST resistance breakpoint indicated by solid black line. Error bars represent 1 SD of the mean, each isolate was tested 3 times.

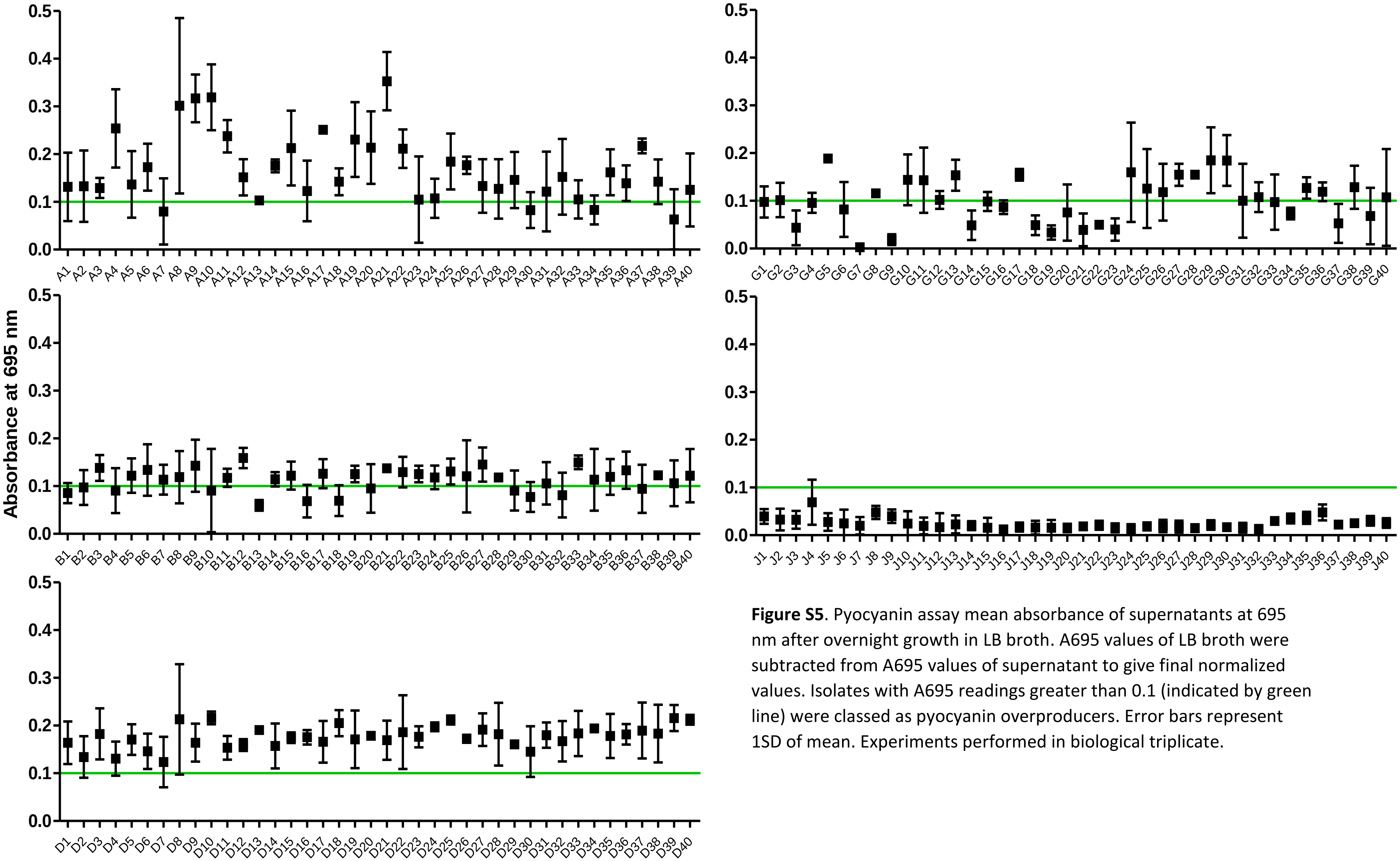

Figure S5. Pyocyanin assay mean absorbance of supernatants at 695 nm after overnight growth in LB broth. A695 values of LB broth were subtracted from A695 values of supernatant to give final normalized values. Isolates with A695 readings greater than 0.1 (indicated by green line) were classed as pyocyanin overproducers. Error bars represent 1SD of mean. Experiments performed in biological triplicate.

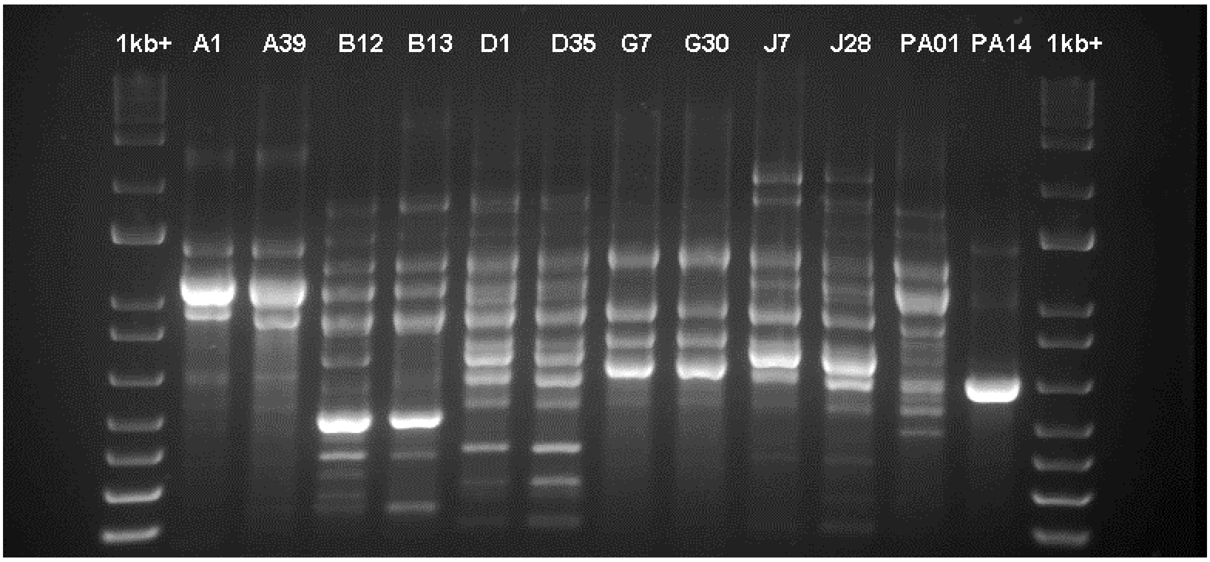

Figure S6. Randomly Amplified Polymorphic DNA (RAPD) PCR products represent boiled genomic DNA from *P. aeruginosa* strains incubated with primer 272 (Sigma, AGCGGGCCAA). Products were run at 70 V for 3 hours on a 1.5% agarose gel with 0.5 X TBE buffer. Ladder is 1 kb+ (Sigma)

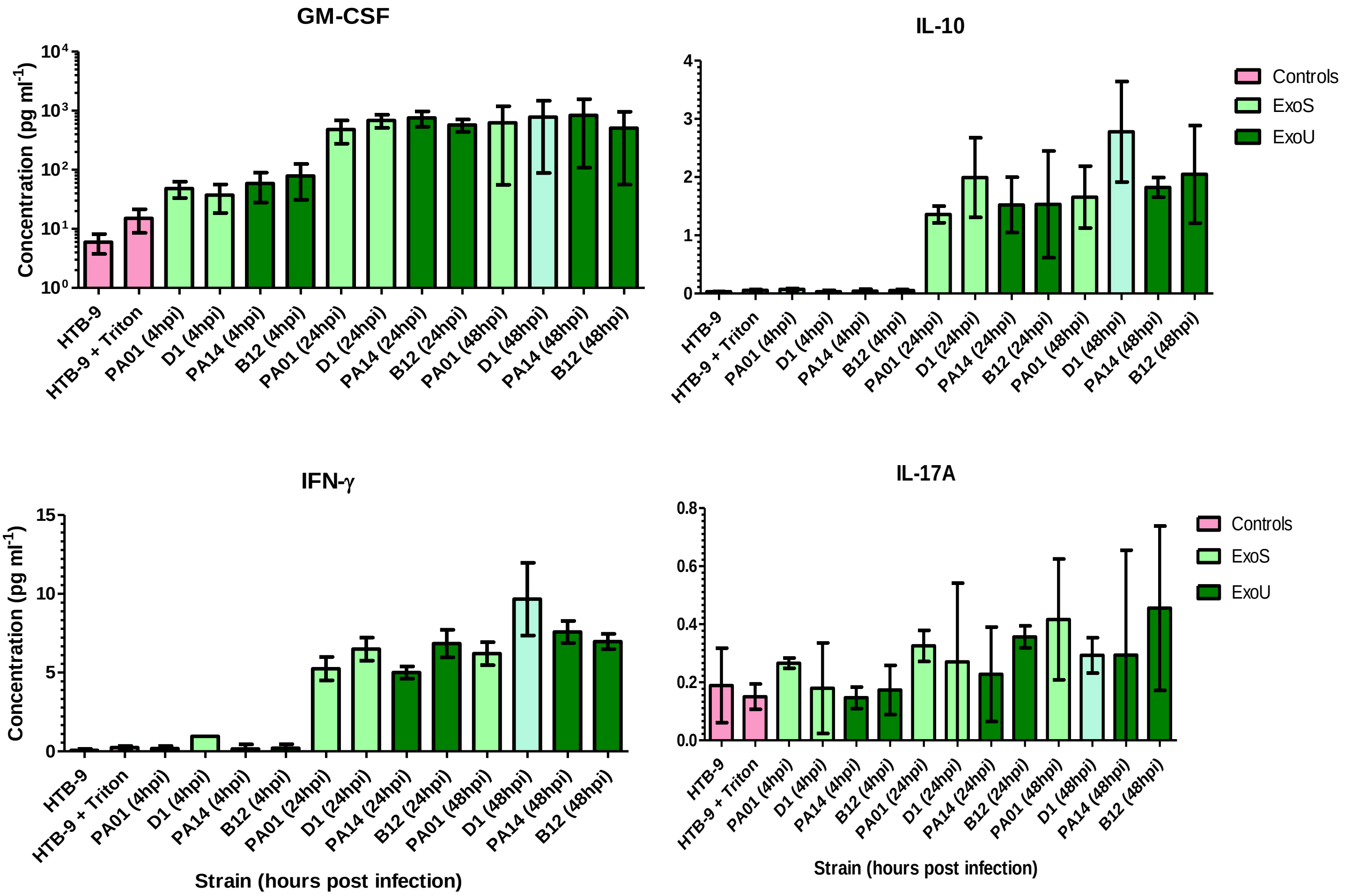

Figure S7. Mean levels of cytokines and pro-inflammatory molecules detected in cell supernatants using the MSD V-plex assay. HTB-9 (5637) human bladder cells were infected with ExoS-type (PA01, D1) and ExoU-type (PA14, B12,) strains for 4, 24 and 48 hours before supernatant was collected and frozen. Experiments performed in biological triplicate. HTB-9 cells treated with Triton X-100 act as a cell death control.

hpi (40 cfu ml^{-1}), 7 hpi (320 cfu ml^{-1}) and 10 hpi (67 cfu ml^{-1}).

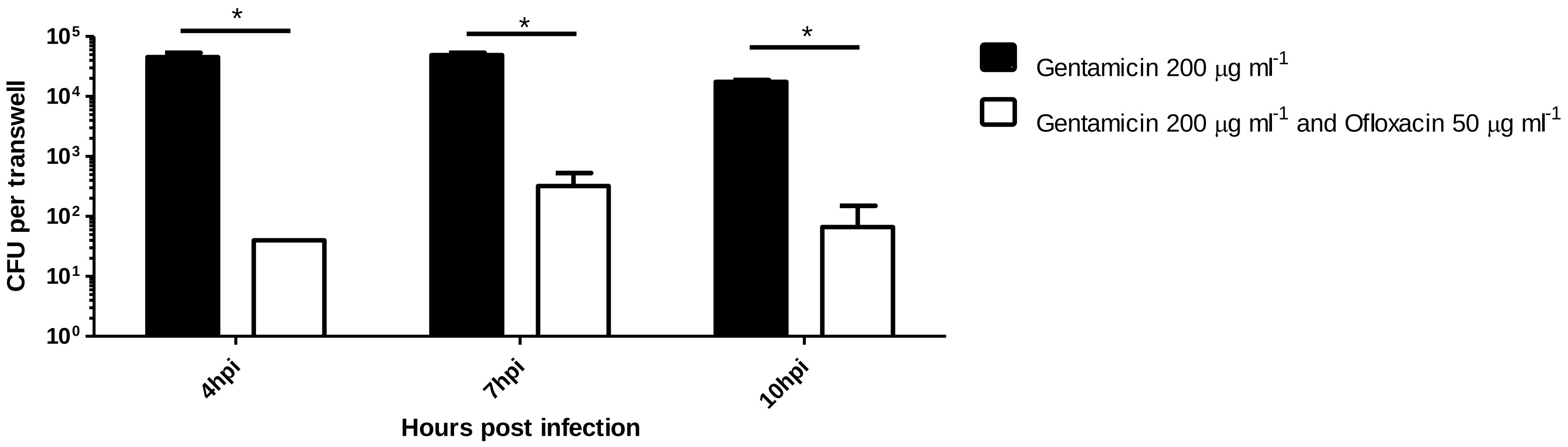

Figure S8. Quantification of *P. aeruginosa* PA01 bacteria isolated at different time points of an *in vitro* invasion assay in 5637 human bladder epithelial cells in transwells. Values represent intracellular bacteria isolated at 4, 7 and 10 hpi following treatment with 200 ug ml^{-1} gentamicin with 50 ug ml^{-1} ofloxacin or just 200 ug ml^{-1} gentamicin. Error bars represent standard deviation of means from technical replicates at each time point and condition (n = 3).
